# Physical and Lifestyle Predictors of Vascular Health in Premenopausal East Asian Women: The Women’s Vascular Health Project

**DOI:** 10.3390/diseases14040144

**Published:** 2026-04-15

**Authors:** Wei Xiong, Fei Tang, Beck Graefe, Ana Raquel Calzada Bichili, Duncan Ryan, Joseph Bonner, Arlette Perry

**Affiliations:** 1 Department of Kinesiology and Sport Sciences, University of Miami, Miami, FL 33146, USA; 2 Miami Veterans Affairs (VA) Healthcare System, Geriatric Research, Education, and Clinical Center (GRECC), Miami, FL 33125-1624, USA; 3 Department of Educational and Psychological Studies, University of Miami, Miami, FL 33146, USA

**Keywords:** cardiovascular risk, arterial stiffness, central adiposity, physical activity, lifestyle, women’s health

## Abstract

**Background/Objectives**: Cardiovascular disease is the leading cause of adult deaths globally and has recently been reported to be on the rise in younger adult women. The present study examined the impact of physical and lifestyle predictors of vascular health in 125 apparently healthy premenopausal East Asian volunteers. **Methods**: Vascular health outcomes included carotid–femoral pulse wave velocity (cfPWV), central augmentation index (cAIx), and mean arterial pressure (MAP). Body composition/anthropometric predictors included total adiposity, visceral adipose tissue (VAT) and skeletal muscle mass (SMM), as well as body mass index (BMI) and waist circumference (WC). Lifestyle predictors included the International Physical Activity Questionnaire (IPAQ) and dietary recall. Multivariate linear regression was used to identify independent predictors of combined vascular health and individual vascular outcome variables. The analysis for independent vascular outcomes was repeated after age stratification (<35 years versus ≥35 years). **Results**: VAT showed a significant multivariate effect on combined vascular health outcomes (*p* = 0.002) and independently contributed to cfPWV (*p* = 0.013). WC positively predicted cAIx (*p* = 0.010) while SMM was inversely related to cAIx (*p* = 0.024). BMI positively predicted MAP (*p* = 0.039) in the multivariate analysis. After age adjustment however, only BMI emerged as a significant independent predictor of both cfPWV (*p* = 0.040) and MAP (*p* = 0.024). Furthermore, WC remained positively associated with cAIx (*p* = 0.042) while SMM remained inversely related to cAIx (*p* = 0.038). After age stratification, IPAQ was inversely related to cfPWV while BMI was positively associated with MAP (*p* = 0.035) in women < 35 years only. However, in older women, total adiposity (*p* = 0.040) and total cholesterol (*p* = 0.011) were both positively, while SMM (*p* = 0.046) was negatively associated with cAIx. **Conclusions**: With the exception of age, VAT was the single best predictor of general vascular health in East Asian women. Independent of age, however, BMI, WC, and SMM significantly contributed to independent vascular outcome measures and in combination with age, substantially add to the prediction of vascular risk. Furthermore, stratifying younger versus older premenopausal women resulted in different associations with independent vascular outcome measures demonstrating that across a large age range of premenopausal women, it is important to consider age in the evaluation of vascular health.

## 1. Introduction

Cardiovascular disease (CVD) remains the leading cause of mortality among adult women worldwide, contributing to nearly one-third of deaths in this population annually [1]. The prevalence of CVD among women under 50 is rising, a trend attributed to adverse lifestyle behaviors and metabolic risk factors that negatively affect cardiovascular health at younger ages [1,2]. Traditionally, premenopausal women are at lower risk for CVD due, in part, to the cardioprotective effects of younger age and estrogen on vascular health [3] and favorable lipid profiles [4]. However, research suggests that premenopausal women are not immune to cardiac risk, as physical factors related to obesity, fat distribution, and lifestyle behaviors such as lack of exercise and poor diet blunt the protective effects of age and premenopausal status [5,6] on heart health [7]. This trend is particularly alarming for Asian women, whose unique genetic, environmental, and cultural factors may negatively interact to elevate CVD risk [8]. East Asian women, specifically Chinese women, represent the largest subgroup of Asian women living in the United States [9], therefore this would be an important group to target regarding vascular health.

Body composition and anthropometric assessments including total adiposity, visceral adipose tissue (VAT), waist circumference (WC) and body mass index (BMI) have been extensively examined in relation to CVD risk [10,11]. However, skeletal muscle mass (SMM) has been far less studied in young Asian women [12]. Furthermore, controversy exists regarding the heterogeneity of cardiac risk predictors among different racial and ethnic groups. For example, in White women, central adiposity has been identified as a strong predictor of arterial stiffness and vascular health [13]. In Black women, however, total adiposity is more closely associated with arterial stiffness [14]. Furthermore, a variety of methods have been used to evaluate vascular health, with some measures possessing stronger relationships with CVD risk than others. In the present study, gold-standard measurements including carotid–femoral pulse wave velocity (cfPWV), central augmentation index (cAIx), and mean arterial pressure (MAP) [15] were used to assess vascular health to provide a more comprehensive assessment of CVD risk.

Lifestyle behaviors are also key determinants of vascular health. Regular physical activity promotes healthy endothelial function, reduces arterial stiffness, and supports optimal blood pressure (BP) regulation through various mechanisms including improved nitric oxide availability and reduced oxidative stress [16,17]. However, it is unclear whether quicker, easier, and less expensive measures of physical activity using self-reported questionnaires can accurately predict vascular health in young Asian women, who remain understudied. Nutritional intake, specifically high consumption of sodium, simple sugars, dietary cholesterol, and saturated/trans fats, comprises dietary behaviors with well-established links to high BP, adiposity, dyslipidemia, and CVD in the general population [18,19,20]. We know of no research examining lifestyle behaviors encompassing physical activity and nutritional intake in a single study addressing vascular health in young Asian women.

Studies of young women across several decades have traditionally been categorized into one “premenopausal” group. For example, Wildman et al. [14] included women spanning 20–40 years in a single premenopausal group and found that anthropometric measures (BMI, WC, hip circumference, and waist-to-hip ratio) were among the strongest predictors of increased arterial stiffness. However, in another study of 20–30-year-old women, Phillips et al. [21] reported central fat distribution to be the best predictor of arterial stiffness, with BMI showing no significant association. These contrasting findings suggest that, even within a homogeneous group of young premenopausal women, body composition predictors of vascular health may vary by age. The present study addresses the contribution of age along with physical and lifestyle predictors of gold-standard vascular outcome measures. By identifying significant predictors of vascular health early on, researchers can develop and implement appropriate intervention strategies to reduce the adverse progression of vascular function and ultimately cardiac risk in this vulnerable population.

## 2. Materials and Methods

### 2.1. Study Design and Population

A total of 125 premenopausal East Asian women between the ages of 20 and 52 completed testing. Participants were recruited from multiple cities across the United States, not solely from a single university and its surrounding area. Recruitment was conducted through flyers, social media, and word of mouth. No satellite sites or remote testing were involved. All vascular assessments were conducted at the university laboratory. All participants were screened using a standard medical history form. Exclusion criteria included a history of (a) hypertension (beta blockers, ACE inhibitors, calcium channel blockers, angiotensin II blockers, diuretics, etc.), (b) diabetes (sulfonylureas, biguanides, SGLT 2 inhibitors, DPP4 inhibitors, GLP-1 agonists, etc.), and/or (c) dyslipidemia (statins, bile acid sequestrants, fibrates, cholesterol absorption inhibitors, niacin, etc.). Additional exclusion criteria encompassed (a) medications known to influence vascular function, (b) use of oral contraceptives, (c) a history of tobacco smoking within the last 12 months, (d) fewer than nine menstrual cycles within the last 12 months, and/or (e) pregnancy.

Written informed consent was obtained from each participant. The study was approved by the Institutional Review Board of the University of Miami (#20180774). Participants were instructed to abstain from exercise for 24 h before testing. They were also instructed to maintain hydration the day before and day of testing. Participants were told to refrain from ingesting caffeine on the day of testing, including the following list of foods: coffee, green/black tea, energy drinks, and chocolate.

### 2.2. Measurement of Physical Characteristics

Height was assessed using a wall-mounted stadiometer (SECA, Hamburg, Germany) and BMI was calculated as BMI = kg/m^2^. A non-invasive body composition analyzer, SECA mBCA 514 (Hamburg, Germany), was used to assess body weight, total adiposity, SMM, and VAT volume [22]. This machine uses bioelectrical impedance analysis (BIA) to determine resistance of an electrical current to fat tissue and lean muscle mass using an octo-polar set of 4 pairs of electrodes that evaluate electrical conductance through all four extremities. Based upon resistance of the tissues to an electrical current, BIA can be used to provide precise and reproducible measurements of various body composition parameters including muscle, fat, and water distribution. In a comparative analysis, the SECA mBCA 514 was correlated with MRI for skeletal muscle mass (CCC = 0.947) [23] and VAT (ICC = 0.949) [24,25]. A Gulick spring-loaded measuring tape (Country Technology Inc., Gays Mills, WI, USA, 67020) was used to measure WC taken midway between the lower margin of the last palpable rib and the top of the iliac crest [22]. For the purposes of this study, anthropometric measurements (BMI and WC) were included under body composition assessments.

### 2.3. Measurement of Vascular Markers of Cardiac Risk

The SphygmoCor XCEL (AtCOR Medical, Wet Ryde, NSW, Australia) uses non-invasive applanation tonometry to analyze central arterial pressure waveforms via pulse wave analysis. This allows the analysis of three different measures of vascular health: cfPWV, cAIx, and MAP. The cfPWV is considered the gold-standard measure of aortic stiffness (vascular health), having been validated against invasive cardiac catheterization procedures for existence of CVD or cardiac risk [15]. During this procedure, participants are asked to lay in a supine position on a treatment table in a quiet environment with controlled temperature (22–24 °C). A tonometer is lightly pressed against the participant’s skin after palpation of the right carotid artery while simultaneously occluding the right femoral artery using a thigh pressure cuff generating central pressure waveforms produced by blood flow velocity. The pulse waves propagated along the arteries during systole are met by reflected waves returning to the heart. Concomitant with pulse transit time and distance traveled by the pulse wave, these measures are used to calculate cfPWV. Pulse transit time was assessed using the “foot-to-foot” method, and wave “feet” were identified with software using intersecting tangent algorithms [15]. By measuring the distance travelled from (a) the carotid sinus pulse wave to the suprasternal notch, (b) the suprasternal notch to the top of the femoral pressure cuff, and (c) the femoral pulse to the top of the femoral pressure cuff, cfPWV can be estimated by the SphygmoCor XCEL software version 1.3.2.18.

The cAIx assesses central aortic pressure by measuring the degree to which aortic waves are reflected, indicating how much extra load is experienced during systole. This offers critical insight into vascular compliancy and the work of the heart [26]. The cAIx is measured using a pressure cuff placed around the right arm which is slightly overinflated to capture the pulse wave. Using a built-in algorithm, the brachial waveform is transformed into a central pressure waveform that mimics the aortic pulse wave. Using this waveform, the device automatically calculates the forward central arterial wave along with any added pressure from the reflected wave to assess the work of the heart during systole. The cAIx is identified by the inflection point, signifying the additional pressure to the heart by the reflected (incident) wave or SBP divided by the pulse pressure (systolic BP (SBP) − diastolic BP (DBP)).

The MAP uses an oscillometric method to identify SBP and DBP according to the standard formula: MAP = DBP + 1/3(SBP − DBP). This calculation, derived from an algorithm of brachial BP, is used to determine central BP, providing an accurate estimate of average arterial pressure during a single cardiac cycle. The MAP provides an assessment of tissue perfusion and overall cardiovascular function [27]. The results for cfPWV, cAIx and MAP, along with pulse pressure and heart rate, are displayed on a special monitor, offering a comprehensive interpretation of the patient’s overall cardiovascular status.

### 2.4. Assessment of Physical Activity

The International Physical Activity Questionnaire (IPAQ) was used to obtain a self-reported measure of physical activity in MET-min/week. The questionnaire comprises seven questions assessing physical activity engagement during the last seven days. A 12-country reliability and validity study indicated that IPAQ questionnaires produced repeatable data, with Spearman’s correlation coefficients clustered around 0.8 for reliability, showing very good repeatability. The criterion validity against the Computer Science and Applications (now Micro Life Technologies, Inc., Shalimar, FL, USA) accelerometer had a median correlation coefficient of approximately 0.30, which is comparable to most other self-report validation studies [28].

### 2.5. Assessment of Dietary Recall

Participants completed two 24 h dietary recalls, one on a weekday and the other on a weekend. This information was used to determine whether dietary factors served as a moderating variable impacting vascular function and/or predictors. During initial screening and prior to arrival at the laboratory, participants were instructed on how to keep an accurate record of all food and beverages consumed within 24 h for one weekday and one weekend day. During laboratory visits, the principal investigator guided participants in recalling the type and amount of all foods and beverages consumed within the last 24 h. Dietary information from both days was analyzed for daily sodium intake, total saturated fat, total cholesterol, and total sugar using the Nutritionist Pro Software, version 7.9 (Axxya Systems, Woodinville, WA, USA), which provides a data base of more than 80,000 ingredients. According to Basiotis [29], a 2-day food log is sufficient to analyze the nutrient composition of foods, given the number of participants enrolled in our study. For each nutrient, the average of the 2-day recall was used for analysis.

### 2.6. Statistical Analyses

Statistical analyses were performed using the IBM SPSS version 29 (SPSS Inc., Chicago, IL, USA), with a significance threshold of *p* < 0.05. Descriptive statistics, including means and standard deviations, were computed for participant characteristics and key outcome variables. Prior to conducting inferential analyses, data were evaluated for normality of distribution using Shapiro–Wilk tests and visual inspection of histograms and Q-Q plots.

Multivariate regression analyses were performed to identify independent predictors of vascular health, with cfPWV, cAIx, and MAP as dependent variables. The predictor variables included five physical (body composition/anthropometric indices) and five lifestyle variables (physical activity and nutritional components). All predictor variables were entered simultaneously into the model to assess their impact on vascular outcomes including cfPWV, cAIx, and MAP simultaneously. Given the large age range of premenopausal women, a second multivariate regression analysis was performed, stratifying by age (≤35 vs. ≥35 years). This was done because premenopausal women in the latter half of their 30s are at a later reproductive age and may possess more subtle hormonal shifts and early ovarian aging than their younger peers [30].

For the multivariate analyses, Wilks’ Lambda, *p*-values, and partial eta-squared (η^2^) were reported to assess the overall model significance and effect sizes. Multicollinearity was assessed using variance inflation factors (VIFs), with values >10 indicating potential concern (Table S1). No multicollinearity was found. Additionally, separate regression coefficients (B) and partial η^2^ values were reported for each predictor of each vascular outcome.

A priori power analysis was conducted using G*Power 3.1 to determine the required sample size for multiple regression. Assuming a moderate effect size (target R^2^ = 0.15, equivalent to Cohen’s f^2^ = 0.18), α = 0.05 (two-tailed), and power (1–β) = 0.80 with ten predictors, our analysis required a total sample of 100 participants. The achieved sample size for the subjects (*N* = 125) exceeded this requirement, suggesting adequate power for the planned analyses.

## 3. Results

### 3.1. Participant Characteristics

Participant characteristics are presented in Table 1. The study sample included 125 premenopausal East Asian women with a median age of 32 years. Anthropometric measurements demonstrated a median height of 163 cm and median weight of 57.1 kg. The BMI values were at the low end of median, 21.68 kg/m^2^, while total adiposity averaged over 30%. Nutritional analyses showed that mean sodium intake, total cholesterol and total sugar exceeded general recommendations of 2300 mg, 300 mg and 50 mg, respectively, set forth by the U.S. Department of Agriculture [31]. Saturated fat intake was within the average range.

### 3.2. Overall Multivariate Linear Regression Test on the Combined Vascular Health Outcomes

The multivariate linear regression model both with and without age adjustment is shown in Table 2. Using this model, VAT was the only predictor having a significant multivariate effect on combined vascular health outcomes (cfPWV, cAIx, and MAP) (Wilks’ λ = 0.880, *p* = 0.002, partial η^2^ = 0.120). However, after adjustment for age, the multivariate effect of VAT was no longer evident (Wilks’ λ = 0.952, *p* = 0.137).

### 3.3. Predictors for Independent Vascular Health Outcomes

In multivariable models without age adjustment (Table 3), VAT was positively associated with cfPWV (B = 0.675, *p* = 0.013, partial η^2^ = 0.053). For cAIx, higher WC was related to higher values (B = 0.637, *p* = 0.010, partial η^2^ = 0.056), whereas higher SMM was related to lower values (B = −0.902, *p* = 0.024, partial η^2^ = 0.044). For MAP, a higher BMI was associated with a higher central pressure (B = 1.075, *p* = 0.039, partial η^2^ = 0.037). No other body composition measure, or any lifestyle predictors, reached statistical significance.

After adjusting for age, BMI emerged as the only independent predictor of cfPWV (B = 0.065, *p* = 0.040, partial η^2^ = 0.037). For cAIx, the positive association with WC (B = 0.470, *p* = 0.042, partial η^2^ = 0.036) and the inverse association with SMM (B = −0.771, *p* = 0.038, partial η^2^ = 0.038) persisted. BMI remained a positive predictor of MAP (B = 1.174, *p* = 0.024, partial η^2^ = 0.044). No other body composition or lifestyle variables reached statistical significance.

### 3.4. Vascular Health Predictors Stratified by Age

In age-stratified multivariable linear regression models (Table 4), distinct predictors of vascular health markers were observed across the two age groups. Among women aged <35 years, higher BMI was independently associated with higher cfPWV (B = 0.104, *p* = 0.002, partial η^2^ = 0.141), whereas higher IPAQ was associated with lower cfPWV (B = −0.000, *p* = 0.030, partial η^2^ = 0.070). For MAP, higher BMI was also associated with higher values (B = 1.038, *p* = 0.035, partial η^2^ = 0.067). No significant predictors were identified for cAIx in this younger group.

Among women aged ≥35 years, higher BF% was associated with higher cAIx (B = 1.466, *p* = 0.040, partial η^2^ = 0.112), whereas higher SMM was associated with lower cAIx (B = −2.220, *p* = 0.046, partial η^2^ = 0.106). In addition, higher average cholesterol intake was positively associated with cAIx (B = 0.029, *p* = 0.011, partial η^2^ = 0.165). There were no significant predictors for any other vascular outcome variables in this older age group.

## 4. Discussion

Recent epidemiological data have revealed an increase in the CVD burden among women under 55 years, marked by higher CVD mortality rates compared to men [32]. Since vascular health encompasses several components vital to cardiac risk, we simultaneously combined three vascular health measures into one model. Not surprisingly, VAT emerged as the best predictor, contributing 12% (*p* = 0.002) of the variance in the combined vascular health measures. This may be due, in part, to the close proximity of VAT to the liver, which increases the amount of free fatty acids, pro-inflammatory cytokines, and chemokines released into circulation, adversely impacting arterial stiffness [33]. However, after adjustment for age, VAT was no longer a significant predictor. This may be related to its positive correlation with age (r = 0.4, *p* < 0.001). In fact, after age adjustment there were no physical or lifestyle predictors of the three combined vascular health measures, signifying much of the variance in vascular health is attributed to age.

Since each of the three outcome measures represent a different feature of cardiovascular health, each variable was evaluated independently. BMI emerged as a significant predictor of cfPWV only after age adjustment. This age-independent “size/hemodynamic load,” although a modest 3.7%, is noteworthy. Asian women possess a higher total adiposity at a given BMI than Caucasian women, which contributes to their risk of CVD and Type 2 diabetes [34,35]. While age may be the driver of VAT in relation to vascular health, BMI encompasses a variety of elements associated with vascular function, including blood volume, wall stress, and inflammation [36]. These may act cumulatively to increase arterial stiffness independent of age. Much of the shared variance among the body composition predictors of cfPWV (e.g., VAT, WC, BF%) are captured by age, leaving less room for independent predictors except for BMI. Our findings in East Asian women contrast with those from the Framingham Study [37], which showed VAT possesses a stronger association with standard CVD risk classifications. However, this study used traditional BP, lipoprotein, and fasting glucose measures as part of their risk assessment and their research was conducted in primarily White populations. Thus, in premenopausal East Asian women, a simple BMI measurement in combination with age accounted for almost 24% of the variance in cfPWV, signifying the collective importance of both measures for arterial stiffness assessment [15]. Upon further analysis, in women <35 years greater IPAQ scores were associated with lower cfPWV, indicating lower arterial stiffness. This contrasts with women ≥35 years and shows that a self-reported activity questionnaire in younger women can be used to identify lifestyle behaviors associated with greater vascular compliance.

Paradoxically, WC but not VAT showed a significant positive association with cAIx. This illustrates the detrimental effect central adiposity may have on intrathoracic pressure augmentation (wave reflection). Since WC encompasses both VAT and subcutaneous adipose tissue, it accounts for a greater total pool of pro-inflammatory markers that may increase total peripheral vascular resistance, peripheral wave reflection, and arterial tone. Collectively, both predictors would be more closely associated with systolic load on the heart rather than central artery stiffness and rigidity. Together, both age and WC contributed to almost 20% of the variance in cAIx, providing an important measure of late-systolic afterload and pulsatile stress on the left ventricle. Our results underscore the age-independent contribution of BMI on cfPWV and WC on cAIx in premenopausal East Asian women.

Few independent studies have examined the contribution of SMM to vascular health in premenopausal women, and to our knowledge no studies have examined this relationship in East Asian women exclusively. Our findings confirmed that a greater SMM was associated with a lower cAIx, illustrating a possible protective effect of SMM on cardiac load. Even after age adjustment, the loss in shared variance with cAIx was <1%, highlighting the independent contribution of skeletal muscle to late systolic afterload and work of the heart. Since muscle enhances insulin sensitivity and reduces inflammation, it may contribute to better metabolic function and vascular health [38,39].

Upon stratification of women by age, the significant inverse relationship between SMM and cAIx was found in the group >35 years. This supports the fact that age may be driving the protective effect of SMM on cAIx. There may also be a change in the hormonal or metabolic milieu driving this relationship that accompanies age [30]. Total adiposity was also related to cAIx in the older group of participants. However, this may be related to the fact that total adiposity places greater work and demands upon the heart. Certainly, a greater sample size with the required statistical power is necessary to further clarify this relationship. This is particularly relevant for Asian women who possess lower SMM than age-matched women of other racial/ethnic groups [40]. They also possess a greater total adiposity at a given BMI level [34]. Similar to WC, the combination of SMM and age accounted for roughly 20% of the variance in systolic load. Given the growing concern surrounding early-onset sarcopenia and its metabolic implications, these results support the integration of resistance training and muscle-strengthening exercises for CVD prevention. Muscle-strengthening exercise may be especially beneficial for those displaying greater central obesity coupled with low-average BMI levels [34,35] and lower SMM [40].

Interestingly, total cholesterol was the only dietary variable showing a positive relationship with cAIx, specifically in women >35 years. Traditionally, CVD is considered an inflammatory disease precipitated by cholesterol deposition, fibrous connective tissue and narrowing of the arterial lumen. However, there is controversy regarding observational studies and lack of causality in findings [41]. Research in guinea pigs and humans has shown that there are responders and non-responders to high cholesterol intakes. For example, some people were non-responders to high cholesterol from daily egg consumption, whereas others showed an increased cholesterol response. However, eggs were not a commonly listed dietary nutrient among our participants and, for the most part, boiled eggs were consumed. Furthermore, total cholesterol is often accompanied by increased free fatty acids known to increase atherogenic LDL- cholesterol and this may confound the effects of cholesterol on heart risk. Participants in our study had average intakes of saturated fat yet higher than average cholesterol intake. Women ≥35 years possessed a mean total cholesterol intake of 400.58 mgs, which is certainly higher than the current recommended level of <300 mgs [31]. In one study of hypercholesterolemic compared to control subjects, higher central BP and cAIx were found in those with greater cholesterol; however, no significant differences in peripheral BP were found between groups [42]. Although the effects of cholesterol on vascular risk remain controversial, cholesterol levels may need to be further evaluated when examining vascular health.

Not surprisingly, BMI exhibited a positive relationship with MAP, indicating the larger the body size, the higher the central BP, independent of age. However, this relationship may have been driven by the larger number of women < 35 years since the relationship between BMI and MAP was only significant in the younger age group. Our findings are consistent with previous research showing excess body weight to significantly contribute to increased BP [43]. For Asian women, BMI is a particularly strong predictor of BP [44]. This can be accounted for by the activation of the renin–angiotensin–aldosterone system and the sympathetic nervous system, which act to increase vascular resistance and blood volume [45]. In a Taiwanese cohort, the impact of obesity on hypertension risk was more pronounced in premenopausal women than men, suggesting that excess adiposity in women may override the CVD protection typically conferred from premenopausal estrogen [46]. There were no significant predictors of MAP when younger versus older premenopausal women were examined independently. Our findings lend support to early weight management interventions in women, particularly culturally sensitive exercise approaches that increase caloric expenditure and reduce excess adiposity in minority women. Asian women typically engage in lower intensity exercise emphasizing rhythmical movements, balance, yoga, and stretching [47]. These activities do not increase caloric expenditure as much as distance-dependent aerobic physical activities. Therefore, aerobic exercise wherein a greater calorie expenditure is expended per unit time may be a preferable exercise strategy for reducing vascular risk before midlife [48]. Public health initiatives suggest walking more than 6000–8000 steps/day to enhance aerobic fitness, mitigate CVD risk [49], and reduce hypertension, the number one cause of CVD [50].

No other lifestyle behaviors predicted markers of vascular health. Results may be related to the fact that our sample was young, relatively healthy and active based upon IPAQ scores. This may have limited the sensitivity to detect significant associations between physical activity and vascular function. Furthermore, there was large variability in dietary intake, as shown by standard deviations that were high for certain nutrients. The high variability highlights weaknesses associated with self-reported dietary intakes. Although food models and virtual images were used to improve accuracy in reporting, most models were limited to standard American foods not typically associated with Asian diets. A large data base featuring over 80,000 items helped to extend nutritional assessments to more diverse cultures; however, among East Asian women, the food base may have been limited. An East Asian investigator familiar with East Asian foods reviewed self-reported intakes with participants to ensure accuracy. However, a greater number of days may be necessary to obtain nutrient intake, especially when using international populations possessing culturally specific dietary intakes. In general, our volunteers consumed moderate amounts of saturated fat, with sodium, cholesterol, and sugar intakes slightly above recommended levels. The relative homogeneity in diet may have reduced the ability to detect diet–vascular associations. Despite our non-significant findings, healthy lifestyle behaviors remain a critical recommendation for adults to reduce risk for CVD [51].

In summary, premenopausal Asian women may possess distinct body composition predictors strategic to vascular health and cardiac risk. Unadjusted for age, VAT was the single best body composition predictor of general vascular health encompassing a combination of all three vascular outcome measures. However, age was the single best predictor of each vascular health measure, capturing most of the shared variance among body composition predictors. Age in combination with BMI accounted for almost one-quarter of the variance in arterial stiffness. In contrast, both WC and SMM emerged as independent predictors of cAIx. These findings indicate that independent of age, higher WC and lower SMM reflect faster wave return, greater pressure from deflected waves, and increased work of the heart during systole. The contribution of each predictor was modest in this sample of relatively young women; however, over time, increases in WC and/or decreases in SMM substantially add to the prediction of chronic load on the heart. Finally, BMI provided a small independent contribution to MAP which, over time, may have significant health implications for increased BP, the number-one risk factor of heart disease [52]. Consequently, targeted lifestyle programs emphasizing structured aerobic physical activity and weight management early on should be utilized to effectively improve vascular health. Furthermore, resistance training may work best to improve SMM and contribute to adverse age-related increases in systolic loading of the heart. Although this study was statistically underpowered to make definitive associations, it appears important to separate younger from older premenopausal women when examining lifestyle predictors of vascular health. Future research should focus on the long-term effectiveness of culturally tailored lifestyle interventions for improving markers of vascular health in premenopausal Asian women.

### Limitations

There are several limitations that should be considered when interpreting the findings. The cross-sectional nature of the data prevents any causal inferences from being drawn. Longitudinal studies are needed to establish the directionality and causality of associations between body composition and lifestyle factors with vascular health outcomes. While all women were premenopausal, hormonal fluctuations that may occur during each cycle were not measured. Estrogen levels are reported to increase during the follicular phase and decrease during the luteal phase. Consequently, vascular function is expected to improve during the follicular phase when estrogen levels are higher and worsen during the luteal phase when estrogen is lower [53]. However, research in this area remains controversial, with one study showing no differences in vascular function between follicular and luteal phases [54], another meta-analysis showing vascular function improvements from the early-late follicular phase [55], and another showing intra-individual differences from cycle to cycle [56]. More definitive research is needed to examine the changes in vascular function across differing phases of the menstrual cycle and its impact on vascular function across several cycles. Certainly, future studies should include cycle phase identification and the assessment of hormonal status when examining vascular health in young women. While the sample of 125 participants exceeded statistical power recommendations, the findings may not be fully generalizable to all premenopausal East Asian women, particularly those from different geographic regions or socioeconomic backgrounds. Although a 2-day food log is considered an indirect, self-reported assessment, it remains vulnerable to underreporting of food intake. Furthermore, the sample showed a high degree of heterogeneity in food intake, with large variability. Therefore, incorporating a 3-day rather than a 2-day food log or adding another week of self-reporting may be necessary to enhance the accuracy of recorded dietary food intake. Although a large data base of >80,000 ingredients was used to extend assessments to more ethnically diverse populations, the data base may not have been representative of East Asian populations. The assessment of physical activity using IPAQ, though widely used, also relied upon self-reported responses, which can introduce bias or inaccuracies in reporting [57]. Therefore, evaluation of aerobic fitness using field tests may provide a more robust indicator of vascular health across large populations. Both IPAQ and 2-day dietary recalls have inherent limitations, being dependent upon recall and self-reporting. Therefore, non-significant findings may reflect weaknesses in the instruments used rather than a true absence of association. Although the use of bioelectrical impedance to evaluate body composition is readily available, less expensive, and radiation-free, it may possess limitations compared to gold-standard evaluations using DEXA or CT scans. While all subjects were premenopausal, stratifying by age may have important implications for identifying contributors to vascular health in premenopausal women. Furthermore, analysis of blood biomarkers including lipids/lipoproteins, insulin/glucose, and pro-inflammatory markers would improve our knowledge of vascular health.

## 5. Conclusions

Our findings showed that VAT emerged as the best predictor of vascular health acting as a possible surrogate for age. Independent of age, however, BMI, WC, and SMM significantly contributed to independent vascular outcomes. In combination with age, the aforementioned variables substantially the prediction of vascular health outcomes. Stratifying younger versus older premenopausal women may yield different associations with vascular outcomes. Therefore, larger sample sizes are needed to examine differences in predictors of vascular health among premenopausal women spanning a large age range. Our findings have important implications for identifying predictors of vascular health early on using readily available measures of body composition, nutrition, and physical assessments. The identification of key contributors to vascular health in premenopausal East Asian women reinforces the need for culturally sensitive interventions that improve vascular function and reduce CVD risk in this vulnerable demographic.

## Figures and Tables

**Table 1:** Participant characteristics of premenopausal Asian women (*N* = 125).

	Median	Q1, Q3
Subject Characteristics		
Age, y	32.00	26.00, 39.00
Height, cm	163.00	159.50, 166.00
Weight, kg	57.10	52.30, 62.80
Systolic Blood Pressure, mmHg	113.00	105.00, 120.00
Diastolic Blood Pressure, mmHg	69.00	65.00, 74.00
Body Composition		
BMI, kg/m^2^	21.68	20.00, 23.15
Waist Circumference, cm	71.12	67.31, 76.20
Body Fat Percentage, %	32.10	27.90, 35.15
Skeletal Muscle Mass, kg	17.00	15.62, 19.23
Visceral Adipose Tissue, L	1.30	1.10, 1.50
Physical Activity		
IPAQ, MET-min/week	1721.00	915.30, 3041.50
Vascular Function		
Carotid-Femoral Pulse Wave Velocity, m/s	6.30	5.80, 6.60
Central Augmentation Index, %	22.00	12.00, 29.00
Mean Arterial Pressure, mmHg	84.00	77.00, 88.00
Nutrition		
Average Sodium, mg	2546.51	1584.39, 3523.99
Average Saturated Fat, g	19.49	13.85, 27.50
Average Cholesterol, mg	345.30	213.47, 502.40
Average Total Sugar, g	51.04	30.91, 72.41

BMI, body mass index; IPAQ, International Physical Activity Questionnaire.

**Table 2:** Multivariate tests for predictors of vascular health.

	Unadjusted by Age				Adjusted by Age	
Predictor	Wilks’ λ	p	$Partial\;\eta^{2}$	Wilks’ λ	p	$Partial\;\eta^{2}$
Age, yrs	-	-	-	0.702	<0.001	0.298
BMI, kg/m^2^	0.956	0.169	0.044	0.947	0.108	0.053
Waist Circumference, cm	0.941	0.078	0.059	0.961	0.221	0.039
Body Fat, %	0.987	0.699	0.013	0.981	0.553	0.019
Skeletal Muscle Mass, kg	0.956	0.169	0.044	0.962	0.230	0.038
Visceral Adipose Tissue, L	0.880	0.002 *	0.120	0.952	0.137	0.048
IPAQ, MET-min/week	0.995	0.913	0.005	0.975	0.428	0.025
Average Sodium, mg	0.989	0.729	0.011	0.988	0.714	0.012
Average Saturated Fat, g	0.959	0.193	0.041	0.967	0.296	0.033
Average Cholesterol, mg	0.991	0.787	0.009	0.989	0.748	0.011
Average Total Sugar, g	0.947	0.108	0.053	0.949	0.121	0.051

BMI, body mass index; IPAQ, International Physical Activity Questionnaire; * *p* < 0.05.

**Table 3:** Body composition and lifestyle predictors of vascular health markers using multivariate linear regression analysis.

		Unadjusted by Age			Adjusted by Age		
	Predictors	B	p	$Partial\;\eta^{2}$	B	p	$Partial\;\eta^{2}$
**cfPWV**	Age	-	-	-	0.041	<0.001 *	0.196
	BMI	0.047	0.180	0.016	0.065	0.040 *	0.037
	WC	0.015	0.258	0.011	0.005	0.688	0.001
	BF%	−0.011	0.547	0.003	−0.002	0.910	0.000
	SMM	−0.013	0.534	0.003	−0.005	0.782	0.001
	VAT	0.675	0.013 *	0.053	0.150	0.566	0.003
	IPAQ	−0.000	0.495	0.004	−0.000	0.150	0.018
	AS	−0.000	0.719	0.001	−0.000	0.845	0.000
	ASF	0.006	0.345	0.008	0.002	0.737	0.001
	AC	−0.000	0.939	0.000	−0.000	0.967	0.000
	ATS	−0.004	0.051	0.033	−0.003	0.055	0.032
**cAIx**	Age	-	-	-	0.693	<0.001 *	0.155
	BMI	−0.067	0.918	0.000	0.246	0.686	0.001
	WC	0.637	0.010 *	0.056	0.470	0.042 *	0.036
	BF%	0.274	0.439	0.005	0.432	0.190	0.015
	SMM	−0.902	0.024 *	0.044	−0.771	0.038 *	0.038
	VAT	4.829	0.339	0.008	−3.997	0.429	0.006
	IPAQ	−0.000	0.870	0.000	0.000	0.435	0.005
	AS	0.001	0.433	0.005	0.001	0.305	0.009
	ASF	−0.037	0.762	0.001	−0.106	0.349	0.008
	AC	0.006	0.342	0.008	0.006	0.286	0.010
	ATS	0.023	0.547	0.003	0.030	0.386	0.007
**MAP**	Age	-	-	-	0.218	0.092	0.025
	BMI	1.075	0.039 *	0.037	1.174	0.024 *	0.044
	WC	0.269	0.165	0.017	0.216	0.267	0.011
	BF%	−0.111	0.691	0.001	−0.061	0.826	0.000
	SMM	−0.265	0.397	0.006	−0.224	0.472	0.005
	VAT	−4.169	0.295	0.010	−6.952	0.106	0.023
	IPAQ	−0.000	0.873	0.000	0.000	0.700	0.001
	AS	0.000	0.669	0.002	0.000	0.620	0.002
	ASF	−0.109	0.255	0.011	−0.131	0.173	0.016
	AC	0.000	0.989	0.000	0.000	0.978	0.000
	ATS	−0.002	0.933	0.000	0.000	0.996	0.000

cfPWV, carotid–femoral pulse wave velocity; cAIx, central augmentation index; MAP, mean arterial pressure; BMI, body mass index; WC, waist circumference; BF%, body fat percentage; SMM, skeletal muscle mass; VAT, visceral adipose tissue; IPAQ, International Physical Activity Questionnaire; AS, average sodium; ASF, average saturated fat; AC, average cholesterol; ATS, average total sugar; * *p* < 0.05.

**Table 4:** Body composition and lifestyle predictors of vascular health stratified by age using multivariate linear regression analysis.

		Age < 35 Years (*n* = 77)			Age ≥ 35 Years (*n* = 48)		
	Predictors	B	p	$Partial\;\eta^{2}$	B	p	$Partial\;\eta^{2}$
**cfPWV**	BMI	0.104	0.002 *	0.141	0.004	0.969	0.000
	WC	0.010	0.426	0.010	−0.021	0.645	0.006
	BF%	−0.015	0.443	0.009	0.030	0.470	0.015
	SMM	−0.016	0.401	0.011	0.019	0.770	0.002
	VAT	0.066	0.808	0.001	0.767	0.388	0.021
	IPAQ	−0.000	0.030 *	0.070	0.000	0.869	0.001
	AS	0.000	0.766	0.001	−0.000	0.770	0.002
	ASF	−0.004	0.554	0.005	0.009	0.497	0.013
	AC	0.000	0.724	0.002	0.000	0.661	0.005
	ATS	−0.003	0.092	0.043	−0.001	0.760	0.003
**cAIx**	BMI	0.604	0.385	0.012	−1.127	0.492	0.013
	WC	0.420	0.123	0.036	1.218	0.108	0.070
	BF%	0.129	0.763	0.001	1.466	0.040 *	0.112
	SMM	−0.549	0.175	0.028	−2.220	0.046 *	0.106
	VAT	−2.978	0.612	0.004	−22.525	0.134	0.061
	IPAQ	−0.001	0.134	0.034	0.000	0.548	0.010
	AS	0.001	0.081	0.046	−0.001	0.601	0.008
	ASF	−0.098	0.496	0.007	−0.436	0.060	0.095
	AC	0.000	0.970	0.000	0.029	0.011 *	0.165
	ATS	0.047	0.269	0.019	0.038	0.571	0.009
**MAP**	BMI	1.038	0.035 *	0.067	2.146	0.235	0.039
	WC	0.178	0.348	0.014	0.127	0.876	0.001
	BF%	−0.127	0.669	0.003	−0.385	0.611	0.007
	SMM	−0.111	0.692	0.002	−1.004	0.399	0.020
	VAT	−5.546	0.179	0.028	−7.090	0.662	0.005
	IPAQ	−0.001	0.168	0.029	0.000	0.659	0.005
	AS	0.001	0.163	0.030	0.000	0.845	0.001
	ASF	−0.115	0.254	0.020	−0.308	0.218	0.042
	AC	−0.000	0.998	0.000	0.000	0.976	0.000
	ATS	−0.010	0.731	0.002	0.033	0.655	0.006

cfPWV, carotid–femoral pulse wave velocity; cAIx, central augmentation index; MAP, mean arterial pressure; BMI, body mass index; WC, waist circumference; BF%, body fat percentage; SMM, skeletal muscle mass; VAT, visceral adipose tissue; IPAQ, International Physical Activity Questionnaire; AS, average sodium; ASF, average saturated fat; AC, average cholesterol; ATS, average total sugar; * *p* < 0.05.

## Data Availability

The data presented in this study are available upon request to the corresponding author.

## Supplementary Materials

The following supporting information can be downloaded at: https://www.mdpi.com/article/10.3390/diseases14040144/s1, Table S1: VIF of covariates.

## Abbreviations

The following abbreviations are used in this manuscript:

CVD	Cardiovascular disease
cfPWV	Carotid–femoral pulse wave velocity
cAIx	Central augmentation index
MAP	Mean arterial pressure
BMI	Body mass index
BP	Blood pressure
VAT	Visceral adipose tissue
BF%	Body fat percentage
SMM	Skeletal muscle mass
WC	Waist circumference
IPAQ	International Physical Activity Questionnaire
