# Supplementary material for: Physical and Lifestyle Predictors of Vascular Health in Premenopausal East Asian Women: The Women’s Vascular Health Project
Source: Diseases. 2026 Apr 15;14(4):144. doi: 10.3390/diseases14040144 (PMC13114829; doi:10.3390/diseases14040144)
Supplement: Supplementary file 1 [file diseases-14-00144-s001.zip › diseases-4222768-supplementary.pdf]

Table S1. VIF of Covariates

**Variable**	**VIF**
Age	1.370
BMI	3.617
Waist	2.376
Body Fat Percentage	3.674
Skeletal Muscle Mass	1.566
Visceral Adipose Tissue	2.497
IPAQ	1.152
Average Sodium	1.411
Average Saturated Fat	1.555
Average Cholesterol	1.659
Average Total Sugar	1.245
